# Neuropeptide Y1 and alpha‐1 adrenergic receptor‐mediated decreases in functional vasodilation in gluteus maximus microvascular networks of prediabetic mice

**DOI:** 10.14814/phy2.13755

**Published:** 2018-07-06

**Authors:** Nicole M. Novielli‐Kuntz, Kent A. Lemaster, Jefferson C. Frisbee, Dwayne N. Jackson

**Affiliations:** ^1^ Department of Medical Biophysics Western University London Ontario Canada; ^2^ Department of Physiology and Pharmacology Western University London Ontario Canada

**Keywords:** Blood flow, functional vasodilation, microcirculation, muscle contraction, norepinephrine, NPY, sympatholysis

## Abstract

Prediabetes is associated with impaired contraction‐evoked dilation of skeletal muscle arterioles, which may be due to increased sympathetic activity accompanying this early stage of diabetes disease. Herein, we sought to determine whether blunted contraction‐evoked vasodilation resulted from enhanced sympathetic neuropeptide Y1 receptor (Y1R) and alpha‐1 adrenergic receptor (*α*1R) activation. Using intravital video microscopy, second‐, third‐, and fourth‐order (2A, 3A, and 4A) arteriolar diameters were measured before and following electrical field stimulation of the gluteus maximus muscle (GM) in prediabetic (PD, Pound Mouse) and control (CTRL, c57bl6, CTRL) mice. Baseline diameter was similar between groups; however, single tetanic contraction (100 Hz; 400 and 800 msec) and sustained rhythmic contraction (2 and 8 Hz, 30 sec) evoked rapid onset vasodilation and steady‐state vasodilatory responses that were blunted by 50% or greater in PD versus CTRL. Following Y1R and *α*1R blockade with sympathetic antagonists BIBP3226 and prazosin, contraction‐evoked arteriolar dilation in PD was restored to levels observed in CTRL. Furthermore, arteriolar vasoconstrictor responses to NPY (10^−13^–10^−8^ mol/L) and PE (10^−9^–10^−5^ mol/L) were greater in PD versus CTRL at higher concentrations, especially at 3A and 4A. These findings suggest that contraction‐evoked vasodilation in PD is blunted by Y1R and *α*1R receptor activation throughout skeletal muscle arteriolar networks.

## Introduction

Peripheral vascular complications associated with type 2 diabetes (Creager et al. 2003) are initiated in the prediabetic state, before manifestation of chronic diabetes disease, where the initiation of vascular dysfunction occurs in the distal microvasculature. (Wiernsperger 1994; Tooke and Goh 1999; Lesniewski et al. 2008; Ellis et al. 2010; Schaefer et al. 2010; Milman and Crandall 2011; Gupta et al. 2012; Reusch et al. 2013).

Prediabetes is a condition of elevated blood glucose, insulin resistance, and hyperinsulinemia that occurs prior to pancreatic *β*‐cell failure and overt type 2 diabetes. Notably, the severity of insulin resistance and elevated plasma insulin has been shown to correlate with the degree of microvascular dysfunction (Jaap et al. 1994, 1997). The microvasculature plays an integral role in regulating blood flow and hematocrit distribution throughout tissues, especially in skeletal muscle, due to its dynamic range across levels of metabolic demand. Importantly, arteriolar microvascular networks not only modulate bulk blood flow in exercising skeletal muscle, but also selectively distribute hematocrit to capillary units supplying active skeletal muscle fibers (Fuglevand and Segal 1997; Murrant et al. 2017). Using intravital video microscopy (IVVM), we previously demonstrated blunted rapid onset vasodilation (ROV) and blood flow to brief tetanic muscle contraction, as well as blunted steady‐state vasodilation and blood flow to sustained rhythmic twitch contractions in branching microvascular networks of prediabetic mice (Novielli and Jackson 2014). However, the mechanisms governing decrements in contraction‐evoked arteriolar responses in prediabetes are not clearly understood.

Under conditions of physical activity, sympathetic nerve activity (SNA) increases and modifies the distribution of cardiac output to sites of highest metabolic demand (Rowell 1993). Heightened SNA can limit skeletal muscle arteriolar vasodilation and concomitant increases in blood flow (Thomas and Segal 2004). In contracting skeletal muscle, direct observations of arterioles confirm that vasodilator and hyperemic responses override elevated sympathetic activation in the active state (Remensnyder et al. 1962). The ability to overcome this effect is termed “functional sympatholysis,” which enables arterioles to increase blood flow and red blood cell flux to capillary beds servicing active muscle fibers (Strandell and Shepherd 1967). Previous studies have shown that type 2 diabetic humans exhibit greater muscle SNA during and following postexercise ischemia of the forearm and during a cold pressor test, compared with healthy controls (Holwerda et al. 2016). Additionally, with the use of microneurography and quantification of plasma catecholamines, studies have demonstrated that hyperinsulinemia, a result of insulin resistance in prediabetes, correlates with elevated SNA (DeFronzo and Ferrannini 1991; Anderson et al. 1992; Berne et al. 1992; Scherrer and Sartori 1997). Heightened sympathetic activity servicing skeletal muscle arterioles may attenuate sympatholysis, decreasing vasodilation and hyperemic potential during muscle contraction in prediabetes (McDaid et al. 1994).

Notably, conditions where heightened SNA is commonly observed, such as aging and the metabolic syndrome, present with impaired skeletal muscle blood flow that has been attributed to enhanced sympathetic *α*‐adrenergic modulation of the vasculature (Frisbee 2004; Jackson et al. 2010; Casey and Joyner 2012). However, it is well established that sympathetic NPY activation of Y1R plays an important role in the skeletal muscle microvascular regulation (Jackson et al. 2004, 2005), thus it is possible that peptidergic perivasvular modulation of arteriolar vasodilation may be responsible for blunted contraction‐evoked arteriolar responses that we recently observed in skeletal muscle (gluteus maximus; GM) arteriolar networks of prediabetic mice (Novielli and Jackson 2014). Previous in vitro experiments using isolated hindlimb arterioles of young prediabetic Zucker Diabetic Fatty (ZDF) rats demonstrated that vasoconstrictor responsiveness to noradrenaline (NA) and endothelin‐1 was enhanced in this cohort compared with controls (Lesniewski et al. 2008). Additionally, our previous in vivo work demonstrated heightened sympathetic neuropeptide Y (NPY) Y1 receptor (Y1R) and alpha‐1 adrenergic receptor (*α*1R) modulation of resting vascular tone in the hindlimb vasculature of prediabetic ZDF rats; where Y1R, *α*1R, and NPY expression was upregulated (Novielli et al. 2012). Collectively, these findings provide evidence of elevated sympathetic nervous system (SNS) influences on vascular control in prediabetes, a condition that may contribute to impaired contraction‐evoked dilation and hyperemia in skeletal muscle microvascular networks of prediabetic mice (Novielli and Jackson 2014).

In this study, we sought to determine whether compromised arteriolar dilation in response to muscle contraction in prediabetes was the result of elevated SNS regulation of the arteriolar microvasculature. Herein, using IVVM and the GM experimental model, the objective of this study was to investigate the effects of Y1R‐ and *α*1R‐mediated arteriolar control on vasodilation to muscle contraction in prediabetic mice across second (2A), third (3A), and fourth order (4A) arterioles. We hypothesize that that blunted arteriolar responses to muscle contraction in prediabetic mice are a result of elevated arteriolar sympathetic Y1R and *α*1R regulation of the microvasculature.

## Research Design and Methods

### Animal care and use

All animal procedures were approved by the Council on Animal Care at The University of Western Ontario (protocol number: 2008‐066). All invasive procedures were performed under *α*‐chloralose and urethane anesthetic, and all efforts were made to minimize animal suffering.

Experiments were performed on male C57BL/6NCrl (7‐ to 8‐week old) and Pound mice (C57BL/6NCrl‐Lepr^db‐lb^/Crl, 7‐ to 8‐week old). The Pound mouse is a model of prediabetes, where these mice exhibit a novel mutation Lepr^db‐lb^ in the leptin receptor gene. The mice become obese by 7 weeks of age, exhibiting hyperinsulinemia, and elevated blood glucose, characteristic of the prediabetic condition in humans (Charles River Laboratories, 2006; Kim and Reaven 2008). As these mice are of C57BL/6 background, the male C57BL/6 mouse served as the control group in this study. Mice were housed in animal care facilities in a temperature (24°C) and light (12 h cycle)‐controlled room and allowed to eat (Purina 5008 chow) and drink water ad libitum. All mice were obtained from Charles River Laboratories (Saint‐Constant, QC, Canada) and housed in animal care facilities for at least 1 week after arrival prior experimentation. Mice were weighed prior to each experiment (PD, 42 ± 1 g; CTRL, 23 ± 1 g; *P *< 0.05). Upon completion of experimental procedures each day, the anesthetized mouse was euthanized with an overdose of *α*‐chloralose and urethane cocktail mix (intraperitoneal injection), and cervical dislocation.

### Measurement of blood glucose and insulin

Mice were fasted (8 h) and blood glucose was measured from a tail vein blood sample (~10 *μ*L) using a Bayer Contour^®^ blood glucose analyzer (Bayer, Toronto, ON, Canada). Fasting blood glucose was greater in PD versus CTRL (12 ± 1 mmol/L vs. 6 ± 1 mmol/L, *P *< 0.05). Prior experimentation, mice were fed ad libitum for at least 2 days following fasting blood glucose measurement. Blood insulin values were not determined, as the blood sample volume necessary to perform the appropriate assay exceeded ethical guidelines for live animals. As such, blood insulin values were obtained from Charles River, where blood insulin levels are elevated in PD (~120 ng/mL) versus CTRL (<10 ng/mL) (Charles River Laboratories, 2006).

### Anesthesia and skeletal muscle preparation

Using an intraperitoneal injection, the mouse was anesthetized with a cocktail of *α*‐chloralose (50 mg/kg) and urethane (750 mg/kg), which was supplemented throughout the experiment via an intraperitoneal catheter upon reflex to a toe pinch. This anesthetic was ideal for these experiments as it leaves autonomic, cardiovascular, and respiratory function intact (Soma 1983). Internal body temperature was monitored via a rectal temperature probe and maintained at 37°C with the use of a heating platform. Surgical procedures were viewed through a stereomicroscope. The neck and backside of the mouse was shaved to remove excess fur. The mouse was placed on its back and a mid‐neck incision was made. A tracheal cannula (PE‐60) was introduced to facilitate spontaneous breathing. The neck opening was then closed using wound clips (Autoclip 9 mm, Becton Dickinson, Franklin Lakes, NJ, USA). The mouse was then placed in the prone position on the heated platform to prepare the GM for IVVM. Under stereomicroscopic guidance, the GM muscle was cut from its origin along the spine and along its rostral and caudal borders (Bearden et al. 2004; Jackson et al. 2010). The muscle flap was reflected away from the mouse, spread evenly onto a transparent Sylgard^®^ (Sylgard 184; Dow Corning, Midland, MI, USA) pedestal to approximate in situ dimensions and pinned to secure edges. The exposed tissue was superfused continuously (4–5 mL/min) with bicarbonate‐buffered physiological salt solution (PSS, 35°C at tissue, pH 7.4) of the following composition (mmol/L): NaCl 137, KCl 4.7, MgSO_4_ 1.2, CaCl_2_ 2, NaHCO_3_ 18, and equilibrated with 5% CO_2_ ⁄ 95% N_2_.

### Intravital video microscopy

Upon completion of microsurgical procedures, the preparation was transferred to the stage of the intravital microscope (Olympus BX51, Olympus, Tokyo, Japan). The preparation was equilibrated with PSS for ~30 min. Microvessels were observed under Kohler illumination using a long working distance condenser (NA = 0.80) and long working distance water immersion objectives (Olympus UMPlanFW: 10′ NA = 0.30; 623× final magnification) with illumination from a 100‐Watt halogen light source. To enhance contrast of the red blood cell (RBC) column, a 450‐nm⁄20‐nm band‐pass filter (450BP20; Omega Optical, Brattleboro, VT, USA) was placed in the light path. The optical image was coupled to a front‐illuminated interline EM CCD camera (Qimaging Rolera E = MC^2^™, Qimaging^©^, Surrey, BC, Canada) and viewed ⁄ stored to a hard drive using specialized imaging software (MetaMorph^®^ 7.6, Molecular Devices Inc., Sunnyvale, CA, USA). Bright‐field video (.tiff) images were collected (15–17 frames per second) under Kohler bright‐field illumination for off‐line analysis of RBC column diameters (Schneider et al. 2012).

Similar to our previous study (Novielli and Jackson 2014), bifurcations at second‐order (2A) to third‐order (3A) arterioles and 3A to fourth‐order arterioles (4A) were chosen for interrogation, as these resistance microvessels are positioned to control the distribution of blood flow within the GM and to the capillaries (Pries et al. 1989; Bearden et al. 2004). One arteriolar tree (2A‐4A) was studied per animal. Following equilibration, a video of the resting (baseline) diameter was taken. Arterioles were then tested for oxygen sensitivity by elevating superfusate O_2_ from 0% to 21% (5% CO_2_, balance N_2_) for 5–8 min to elicit vasoconstriction. Equilibration with 5% CO_2_–95% N_2_ was restored for the duration of experimental procedures. Changes in arteriolar diameter were evaluated in response to brief maximal tetanic contractions at 100 Hz as well as 30 sec of rhythmic muscle contractions (see Skeletal Muscle Contractions). For these experiments, each muscle preparation underwent both contraction protocols with the order randomized across experiments. At the end of each day's procedures, maximum arteriolar diameter was recorded by adding sodium nitroprusside (SNP, 10 *μ*mol/L) to the superfusate (Bearden et al. 2004; VanTeeffelen and Segal 2006; Jackson et al. 2010). It was determined, however, that vasodilation of PD 2A and 3A to SNP treatment was less than that of CTRL arterioles. Responses of PD arterioles to SNP were then tested in the presence of sympathetic antagonists BIBP3226 (100 nmol/L, Y1R antagonist) and prazosin (100 nmol/L, *α*1R antagonist). This was performed to determine whether enhanced arteriolar sympathetic receptor activation contributes to decreased vasodilation to SNP in PD.

### Skeletal muscle contractions

Contractions of the GM were evoked using electrical field stimulation (EFS). For this purpose, wire electrodes (90% Pt–10% Ir; diameter, 250 *μ*m) were positioned in the superfusion solution on either side of the exposed muscle. Monophasic pulses (0.1 msec) were delivered at 10 V through a stimulus isolation unit (SIU5; Grass Technologies; Quincy, MA, USA) driven by a square wave stimulator (S48, Grass Technologies; Quincy, MA, USA). Our experiments and previous work have shown that this voltage elicits reproducible contractions of the GM and of arteriolar responses for the duration of an experiment (Jackson et al. 2010; Novielli and Jackson 2014). In control experiments, addition of 10 *μ*mol/L *d*‐tubocurarine (nicotinic cholinergic receptor antagonist) inhibited muscle contraction to EFS, confirming that muscle contraction was a result of motor nerve activation and not direct depolarization of skeletal muscle cells (Jackson et al. 2010; Novielli and Jackson 2014).

### Tetanic contraction and rapid onset vasodilation

A brief maximal tetanic contraction at 100 Hz was used to evoke ROV in each experimental group. Arteriolar dilations were evoked for stimulus train durations of 400 and 800 msec, with the order randomized across experiments. The arteriole consistently returned to the initial resting baseline with 2–3 min of recovery between contractions. As tissue displacement occurred during tetanic contraction, diameter was measured preceding each stimulus (resting baseline) and immediately following contraction with a delay of ~2 sec that reflected the time the muscle is contracted and field of view out of focus, and the time required to refocus the field of view.

### Rhythmic contraction and steady‐state vasodilation

As the nature of vasodilatation can vary with the pattern of muscle fiber activation (VanTeeffelen and Segal 2000; Murrant 2005), vasomotor responses to 30 sec of rhythmic contractions at 2 and 8 Hz (in randomized order) were also evaluated in each experimental group. Stimulation at these frequencies evoked unfused twitch contractions (Bearden et al. 2004). Following each 30‐sec period of rhythmic twitch contractions, resting baseline was reestablished consistently within 5 min. Arteriolar diameter was determined preceding contractile activity and following the 30‐sec contraction period.

### Muscle contraction experimental conditions

Arteriolar vasodilatory responses to tetanic and rhythmic contraction were first evaluated under control conditions, where PSS was superfused over the GM. Upon establishing differences in vasodilatory responses between CTRL and PD, we sought to determine whether this difference was attributed to alterations in peripheral sympathetic arteriolar activation. As such, we blocked Y1R, *α*1R, and Y1R+ *α*1R with the addition of BIBP3226 (100 nmol/L) and prazosin (100 nmol/L) (TOCRIS, Bristol, UK) in the superfusate. Concentrations did not affect the resting baseline diameter (Table 1). Sympathetic antagonist concentrations used were determined based on the ability to abolish arteriolar constriction elicited by superfusion of 10^−8^ mol/L NPY and 10^−5^ mol/L phenylephrine (PE). Conversely, in an effort to attenuate arteriolar responses to muscle contraction in CTRL, we carried out a series of contraction experiments where Y1R, *α*1R, and Y1R+ *α*1R were activated using NPY (10^−11^ mol/L) and PE (10^−8^ mol/L). Agonist concentrations were determined based on the ability to blunt arteriolar responses to muscle contraction, despite no observable change in baseline arteriolar diameter (Table 1). Agents were added to superfusion solution to working concentrations and allowed to equilibrate with the tissue. The order of drug perturbations was randomized.

**Table 1:** Baseline diameter of gluteus maximus arterioles following mild Y1R and *α*1R activation and inhibition in CTRL and PD, respectively

	CTRL				PD			
	PSS	NPY	PE	NPY + PE	PSS	BIBP3226	Prazosin	BIBP3226 + Prazosin
2A	22 ± 1	22 ± 1	21 ± 1	20 ± 1	20 ± 1	20 ± 1	21 ± 1	20 ± 1
3A	13 ± 1	14 ± 1	15 ± 1	14 ± 1	13 ± 1	13 ± 1	14 ± 1	13 ± 1
4A	8 ± 1	7 ± 1	8 ± 1	8 ± 1	7 ± 1	8 ± 1	7 ± 1	7 ± 1

Values are mean ± SEM. CTRL, control, *n* = 5–6; PD, prediabetic, *n* = 5–10.

### Arteriolar reactivity to sympathetic Y1R and *α*1R agonists

Vasoconstrictor responses of CTRL and PD 2A, 3A, and 4A to sympathetic Y1R and *α*1R agonists NPY (Y1R agonist, 10^−13^–10^−8^ mol/L) and PE (*α*1R agonist, 10^−9^–10^−5^ mol/L) were investigated. The order of NPY and PE drug‐range applications were performed in random order for each experiment, where resting diameter was allowed to recover to baseline after each set of drug perturbations. Baseline diameter was recorded prior to the addition of drug to PSS. At each concentration of NPY or PE, arteriolar diameter was allowed to plateau for 5 min and a video was recorded before the next increment in drug dose. Working concentrations of drugs were prepared fresh on day of experiment, and diluted in PSS. Arteriolar vasoconstrictor responses were determined from the difference between diameter measures taken prior drug perturbations (baseline diameter) and diameter at each drug concentration of NPY or PE.

### Statistical analyses and data presentation

Data were analyzed using Sigmastat (Systat Software Inc, San Jose, CA, USA) and differences were accepted as significantly different at *P *<* *0.05. In order to compare the effect of sympathetic antagonists on PD arteriolar responses to GM tetanic and steady‐state contractions, one‐way analysis of variance within each stimulus level was performed using Dunnett's post test to compare all conditions with the CTRL condition. In order to compare the effect of sympathetic agonists on CTRL arteriolar responses to GM tetanic and steady‐state contractions, one way analysis of variance within each stimulus level was performed using Dunnett's post test to compare all conditions with the PD condition. Differences between CTRL and PD responses within each concentration of NPY or PE were compared using unpaired *t*‐tests. Tabular data were also analyzed using unpaired *t*‐tests. Summary data are presented as mean values ± SE, unless otherwise stated.

## Results

### Baseline arteriolar diameter, O_2_ response, and vasodilator responses to sodium nitroprusside in control and prediabetic mice

Baseline diameters among 2A, 3A, and 4A were similar between CTRL and PD (Table 1). Arteriolar constriction in response to elevating PSS O_2_ to 21% was also similar between groups for 2A (−6 ± 1 *μ*m), 3A (−4 ± 1 *μ*m), and 4A (−2.4 ± 0.2 *μ*m). Maximal arteriolar diameter elicited by 10 *μ*mol/L SNP was attenuated 20 ± 3% and 24 ± 4% in PD 2A and 3A, respectively, versus CTRL (Table 2, *P *< 0.05). Maximal dilation at 4A was similar between groups. Upon blocking sympathetic receptors with BIBP3226 (Y1R antagonist; 100 nmol/L) and prazosin (*α*1R antagonist; 100 nmol/L) during simultaneous SNP (10 *μ*mol/L) superfusion, maximal vasodilatory responses in PD 2A and 3A recovered to CTRL levels (Table 2).

**Table 2:** Maximal diameter responses of gluteus maximus arterioles to sodium nitroprusside with and without sympathetic receptor blockade

	Maximal dilation CTRL (SNP; 10 *μ*mol/L)	Maximal dilation PD (SNP; 10 *μ*mol/L)	Maximal dilation PD + sympathetic blockade (SNP; 10 *μ*mol/L, and BIBP3226 + prazosin; 100 nmol/L)
2A	41 ± 1	33 ± 1^a^	38 ± 2
3A	29 ± 1	22 ± 1^a^	27 ± 2
4A	18 ± 1	17 ± 1	19 ± 1

Values are mean ± SEM. CTRL, control, *n* = 6‐13; PD, prediabetic, *n* = 3‐12.

a *P *< 0.05 versus CTRL.

### Rapid onset vasodilation and sympathetic receptor blockade in prediabetic mice

**Figure 1:**
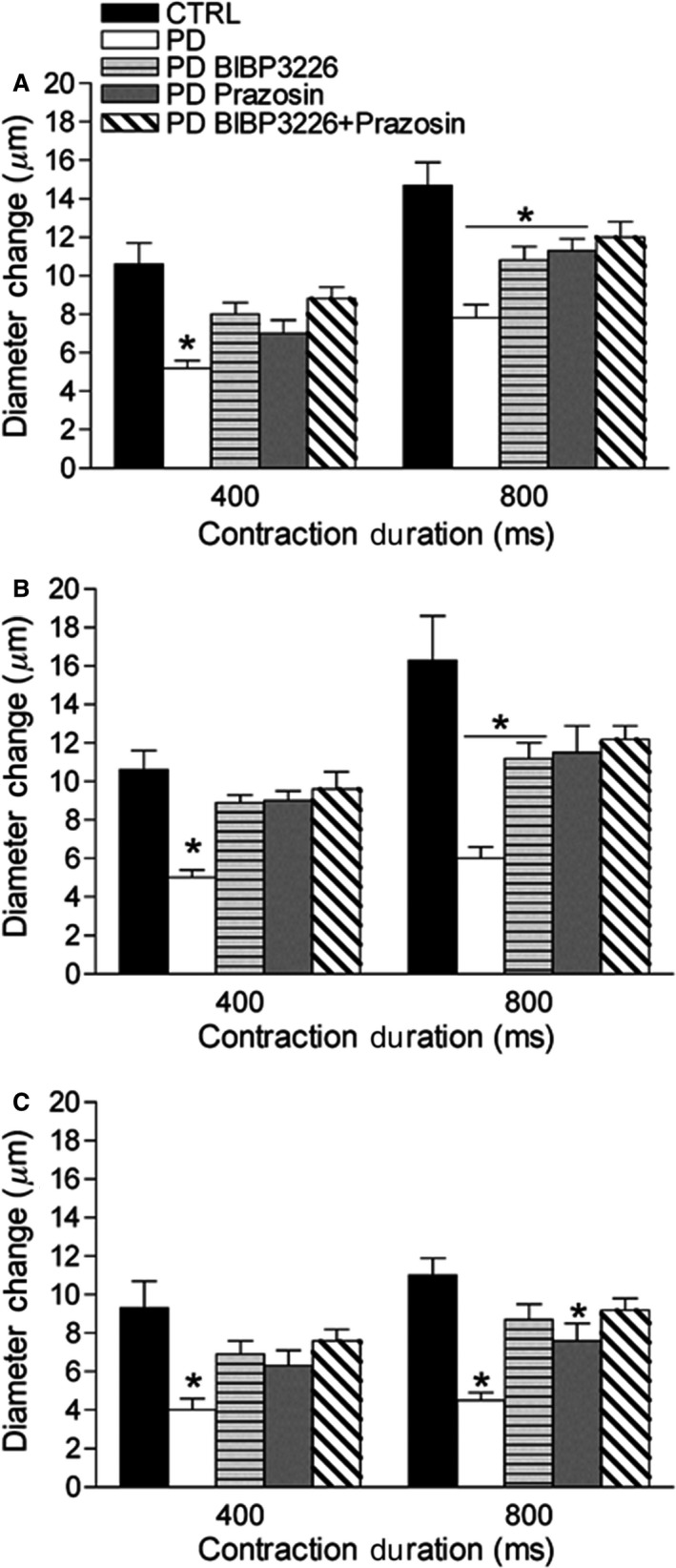
Arteriolar responses to brief maximal tetanic contraction, with and without sympathetic receptor blockade. Data (mean ± SE) are presented as maximum arteriolar diameter responses of 2A (A), 3A (B), and 4A (C) in CTRL (*n* = 6–12) and PD (*n* = 6–11) following 400 and 800 msec tetanic contraction durations, with and without localized sympathetic receptor antagonism (PD). *Different from CTRL,* P* < 0.05. CTRL, control; PD, prediabetic.

Following 400 and 800 msec tetanic contractions, ROV in PD was blunted by 51 ± 6% and 47 ± 6% in 2A, 52 ± 6% and 62 ± 6% in 3A, and 58 ± 9% and 59 ± 6% in 4A, respectively, (Fig. 1, *P *< 0.05). In response to 400 msec contraction, independent Y1R, *α*1R and combined Y1R+ *α*1R blockade normalized ROV in PD similar to CTRL across arteriolar orders (Fig. 1). Following 800 msec contraction, combined Y1R+ *α*1R blockade restored 2A ROV responses of PD to CTRL levels (Fig. 1A); whereas, independent Y1R and *α*1R partially recovered PD 2A ROV responses to CTRL levels (Fig. 1A, *P *< 0.05). For 3A, independent *α*1R and combined Y1R+ *α*1R blockade in PD recovered ROV responses to CTRL levels (Fig. 1B), where independent Y1R blockade in PD only partially recovered ROV to CTRL levels (Fig. 1B, *P *< 0.05). In 4A, independent Y1R and combined Y1R+ *α*1R blockade restored ROV to that of CTRL (Fig. 1C), where independent *α*1R blockade in PD only partially recovered ROV to CTRL levels (Fig. 1C, *P *< 0.05).

### Steady‐state vasodilation to rhythmic contraction and sympathetic receptor blockade in prediabetic mice

**Figure 2:**
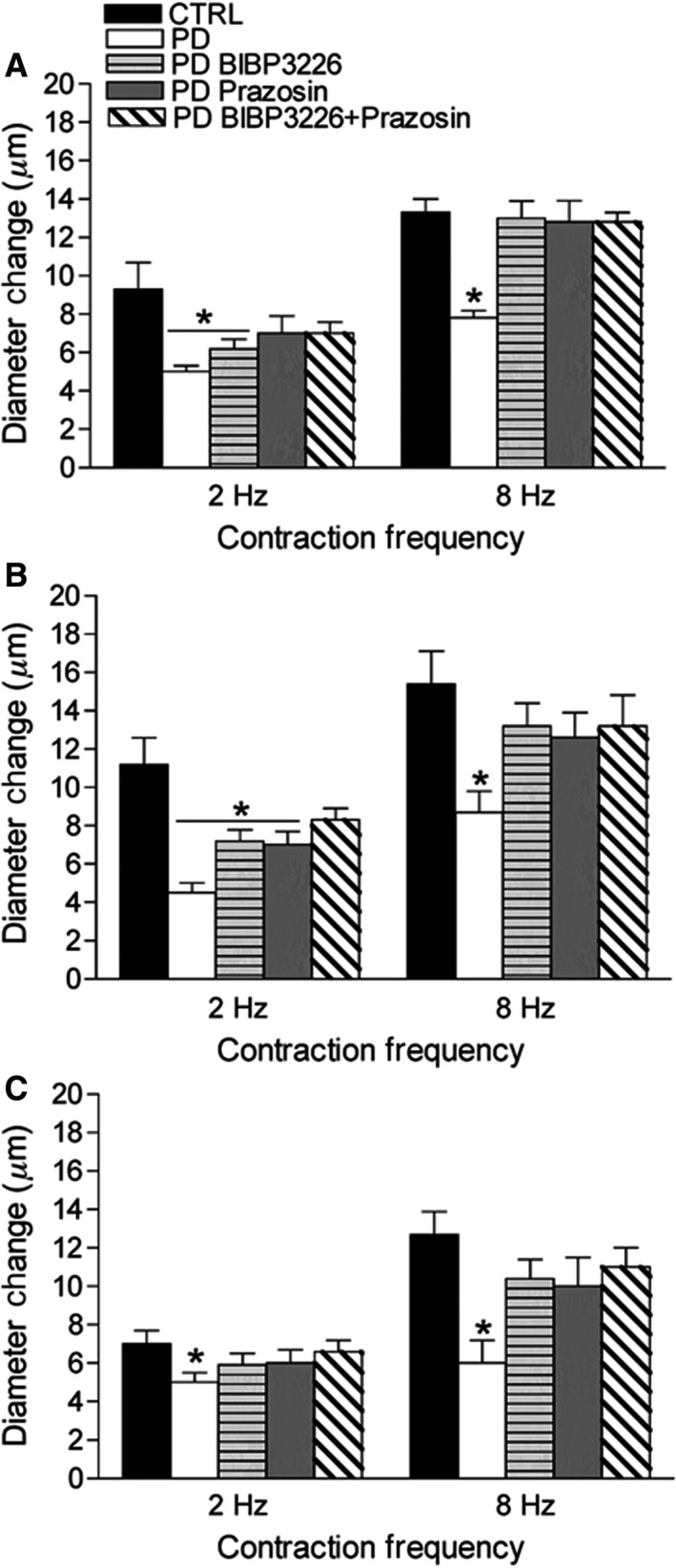
Arteriolar responses to rhythmic twitch contractions, with and without sympathetic receptor blockade. Data (mean ± SE) are presented as maximum arteriolar diameter responses of 2A (A), 3A (B), and 4A (C) in CTRL (*n* = 6–12) and PD (*n* = 6–11) following 30‐sec of 2 and 8 Hz rhythmic twitch contractions, with and without localized sympathetic receptor antagonism (PD). *Different from CTRL,* P* < 0.05. CTRL, control; PD, prediabetic.

Following 2 and 8 Hz rhythmic contractions, vasodilatory responses of PD were blunted by 46 ± 9% and 47 ± 7% in 2A, 60 ± 7% and 44 ± 9% in 3A, and 32 ± 11% and 53 ± 11% in 4A, respectively, (Fig. 2, *P *< 0.05). Following 2 Hz rhythmic contraction, dual sympathetic receptor blockade effectively restored arteriolar responses of PD to CTRL levels at 2A and 3A, where independent Y1R and *α*1R blockade did not (Fig. 2A and B, *P *< *0.05*). Arteriolar responses of 4A in PD were restored by all sympathetic receptor blockade conditions to levels similar to CTRL (Fig. 2C). In contrast, following 8 Hz rhythmic contraction, all conditions of sympathetic receptor blockade in PD recovered arteriolar responses of PD to CTRL levels across all arteriolar orders (Fig. 2, *P *< 0.05).

### Contraction‐evoked dilation and sympathetic receptor activation in control mice

**Figure 3:**
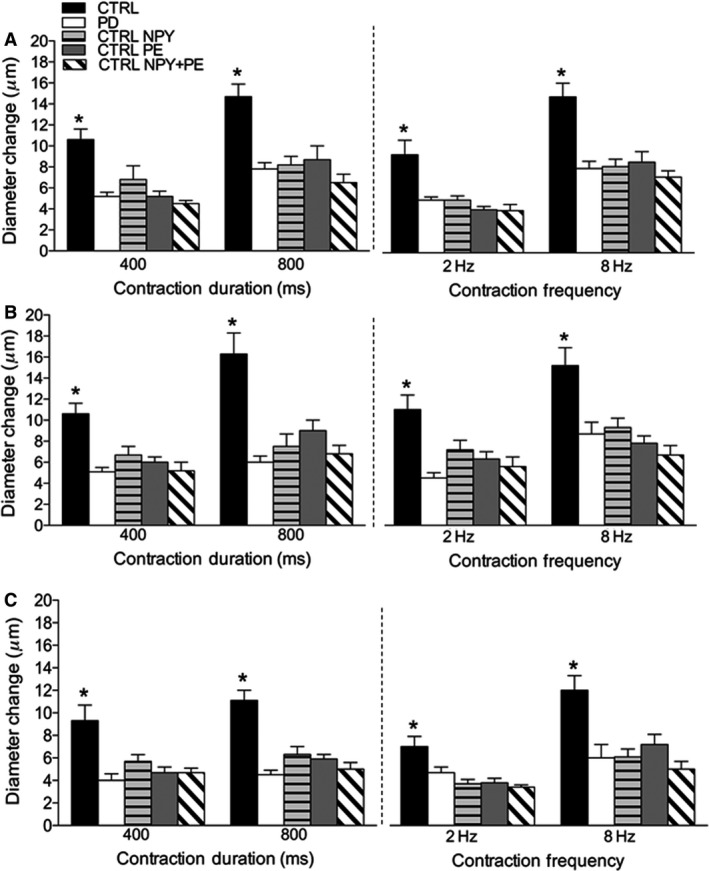
Effects of mild sympathetic Y1R and *α*1R activation on contraction‐evoked arteriolar dilation in CTRL. Data (mean ± SE) are presented as maximal arteriolar dilation in response to a brief maximal tetanic contraction (left panels) and 30‐sec of rhythmic twitch contractions (right panels) of 2A (A), 3A (B), and 4A (C) in CTRL (*n* = 6–12) and PD (*n* = 6–11). NPY (Y1R agonist), PE (*α*1R agonist) and NPY + PE (dual Y1R and *α*1R activation) were added to the superfusion of CTRL. *Different from PD,* P* < 0.05. CTRL, control; PD, prediabetic.

To mimic blunted arteriolar responses to brief tetanic and sustained rhythmic muscle contraction observed in PD, we tested the effects of mild sympathetic receptor activation on rapid onset and steady‐state vasodilation in CTRL. Addition of Y1R and *α*1R agonists (NPY, PE, and NPY+PE) to the superfusate solution during 400 and 800 msec tetanic contraction, and 2 and 8 Hz rhythmic contractions decreased arteriolar responses in CTRL 2A, 3A, and 4A to levels observed in PD (Fig. 3, *P *< 0.05).

### Arteriolar constriction to sympathetic Y1R and *α*1R activation

**Figure 4:**
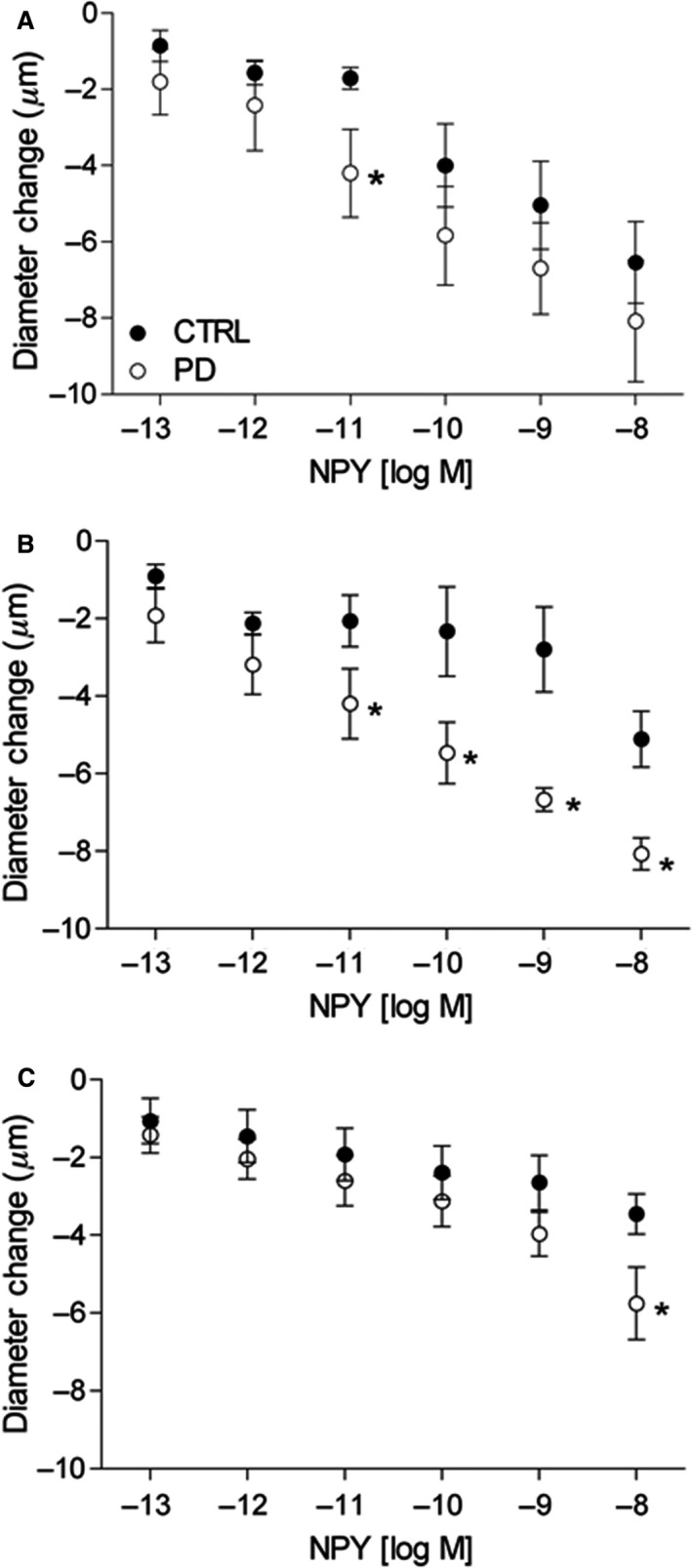
Vasoconstriction of gluteus maximus arterioles in response to topical application of NPY. Data (mean ± SE) are presented as 2A (A), 3A (B), and 4A (C) vasoconstrictor responses to topical application of increasing concentrations of NPY (Y1R agonist) in CTRL (*n* = 5–9) and PD (*n* = 5–7). *Different from CTRL within drug concentration, *P* < 0.05. CTRL, control; PD, prediabetic.

To compare arteriolar sensitivity to sympathetic receptor activation in PD versus CTRL, concentration‐dependent arteriolar responses to Y1R and *α*1R activation were evaluated in branching arteriolar trees (i.e., 2A, 3A, and 4A). Increasing concentrations of Y1R agonist NPY (10^−13^–10^−8^ mol/L) led to progressive decreases in arteriolar diameter in both CTRL and PD (Fig. 4). For 2A, vasoconstrictor responses to NPY were greater in PD versus CTRL, only at NPY 10^−11^ mol/L (Fig. 4A, *P *< 0.05). However, in 3A, vasoconstrictor responses were greater in PD versus CTRL, for NPY concentrations of 10^−11^–10^−8^ mol/L (Fig. 4B, *P *< 0.05). In 4A, vasoconstrictor responses were similar between groups for 10^−13^–10^−9^ mol/L NPY; however, at the highest concentration of NPY (10^−8^ mol/L), vasoconstriction in PD was greater than CTRL (Fig. 4C, *P *< 0.05).

**Figure 5:**
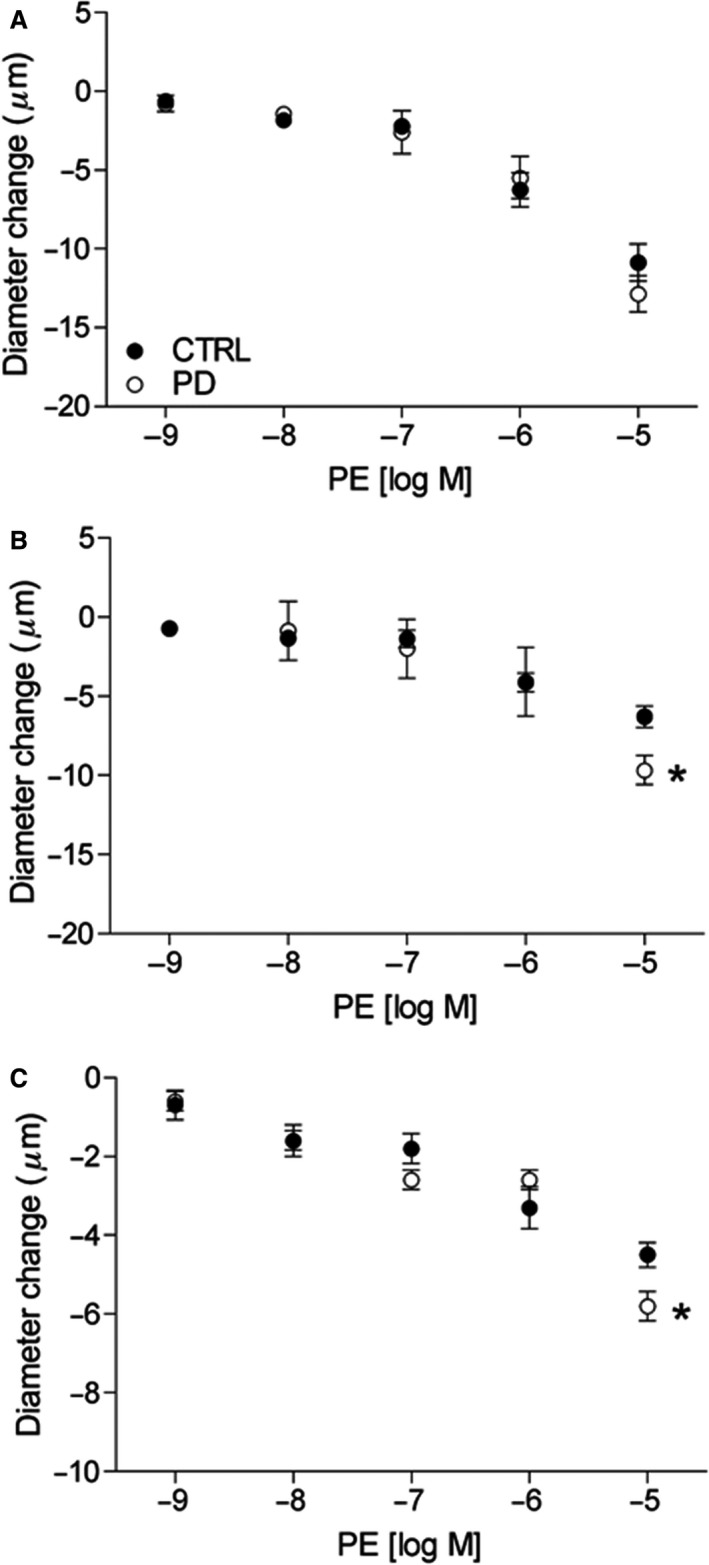
Vasoconstriction of gluteus maximus arterioles in response to topical application of PE. Data (mean ± SE) are presented as 2A (A), 3A (B), and 4A (C) vasoconstrictor responses to increasing doses of PE (*α*1R agonist) in CTRL (*n* = 5–9) and PD (*n* = 5–7). *Different from CTRL within drug dose, *P* < 0.05. CTRL, control; PD, prediabetic.

Increasing concentrations of *α*1R agonist PE (10^−9^–10^−5^ mol/L) also led to progressive decreases in arteriolar diameter in both CTRL and PD (Fig. 5). Vasoconstrictor responses of 2A were similar between groups for all PE concentrations (Fig. 5A). For 3A and 4A, vasoconstrictor responses of CTRL and PD were similar for PE concentrations 10^−9^–10^−6^ mol/L, but greatest in PD at 10^−5^ mol/L versus CTRL (Fig. 5B and C; *P *< 0.05).

## Discussion

The current data provide novel insight regarding the mechanisms governing compromised contraction‐evoked arteriolar dilation in the Pound Mouse model of prediabetes. Herein, we have shown for the first time that prediabetes promotes peptidergic and adrenergic dysregulation across branching arteriolar networks in contracting skeletal muscle. Data from this study are in congruence with our previous work, in which we identified heightened sympathetic (Y1R and *α*1R) regulation of vascular tone and blood flow in hindlimb muscle of prediabetic ZDF rats under baseline (resting) conditions (Novielli et al. 2012).

In the Pound Mouse model of prediabetes, the observed deficits in contraction‐evoked arteriolar dilation in skeletal muscle appears to be mediated by modest activation of Y1R and *α*1R, as sympathetic receptor blockade (with topical application of BIBP3226 and prazosin) in PD recovered contraction‐evoked vasodilator responses to CTRL levels. Additionally, arteriolar vasoconstrictor responsiveness to topical application of sympathetic receptor agonists (i.e., NPY and PE) was up to twofold greater in PD versus CTRL, most notably at higher concentrations and with the greatest differences being observed in responses to NPY.

### Sympathetic Y1R‐ and *α*1R‐mediated effects on contraction‐evoked arteriolar vasodilation in prediabetic mice

Rapid onset vasodilation results in an immediate hyperemic response elicited within seconds of muscle contraction at exercise onset. This near instantaneous vascular response has been well established in humans and within animal microcirculatory models (Corcondilas et al. 1964; Marshall and Tandon 1984; Shoemaker et al. 1998; Mihok and Murrant 2004; VanTeeffelen and Segal 2006; Armstrong et al. 2007; Kirby et al. 2007; Jackson et al. 2010), and is a conserved response in initiating rest‐to‐exercise transitions to match metabolic demand. In the current study, and congruent with previous work, we consistently demonstrated blunted arteriolar ROV responses of ~50% or greater following brief tetanic muscle contraction in the GM of prediabetic mice, with no notable differences in baseline arteriolar diameter. Superfusion of the GM with the sympathetic Y1R antagonist BIBP3226 and *α*1R antagonist prazosin restored attenuated ROV responses of PD to levels observed in CTRL. Interestingly, without modification of baseline arteriolar diameter, mild activation of Y1R and *α*1R with NPY and PE during tetanic contraction blunted arteriolar dilation in CTRL to levels observed in PD. These findings suggest that altered levels of arteriolar vascular smooth muscle cell (VSMC) Y1R and *α*1R activation may impinge on existing dilatory mechanisms responsible for ROV in skeletal muscle microvasculature of prediabetic mice. Past studies investigating skeletal muscle microcirculation in the hamster cremaster muscle have demonstrated a contributing role of potassium and adenosine to ROV responses elicited by brief tetanic contractions (Armstrong et al. 2007; Ross et al. 2013). In human studies, potassium, as well as nitric oxide and prostaglandins have been shown to play a role in the ROV response (Crecelius et al. 2013). Whether increased Y1R and *α*1R activation in prediabetes affect such vasodilatory mechanisms remains to be investigated.

In contrast to brief tetanic contraction, sustained rhythmic muscle contraction evokes a progressive increase in arteriolar diameter and blood flow based on the metabolic demands of the tissue (Bockman 1983; Armstrong and Laughlin 1985; Mohrman and Regal 1988). Studies investigating mechanisms of sustained vasodilation observed during repeated muscle contractions have identified local vasoactive metabolites (e.g., prostaglandins, epoxyeicosatrienoic acid, ATP, and nitric oxide) from skeletal muscle tissue and the vasculature that contribute to this response (Clifford and Hellsten 2004; Saltin 2007; Nyberg et al. 2013). As these signaling events differ from those involved in ROV (Haddy and Scott 1975; Wunsch et al. 2000; Clifford and Hellsten 2004), it was not known whether decrements in contraction‐evoked steady‐state dilation, previously demonstrated in PD (Novielli and Jackson 2014), were a result of sympathetically mediated vasoconstriction. In the current study, there was up to a 60% reduction in steady‐state vasodilation following 30 sec of rhythmic twitch contractions in PD. Upon sympathetic Y1R and *α*1R blockade, arteriolar vasodilatory responses of PD were restored to levels of CTRL. All sympathetic antagonist conditions were especially effective in restoring PD vasodilatory responses following 8 Hz contractions. Activation of arteriolar Y1R and *α*1R in CTRL promoted decreases in contraction‐evoked vasodilatory responses, which resembled responses of PD. These findings further demonstrate that increased levels of arteriolar Y1R and *α*1R activation in prediabetes can restrain vasodilatory responses regardless of the nature of contractile activity, decreasing sympatholytic potential of the microcirculation.

Despite the observed “gain of function,” blockade of Y1R or *α*1R independently did not always restore contraction‐evoked vasodilatory responses of PD for all stimulation conditions and arteriolar orders. This may have resulted due to prevailing vasoconstrictive effects of the active (nonantagonized) sympathetic receptor(s) during independent Y1R or *α*1R ‐blockade. For example during application of BIBP3226, only NPY's effects are blocked and the effects of NA prevail and vice versa. Furthermore, there could be outstanding microvascular complications accompanying sympathetic arteriolar dysregulation in prediabetes; for example, decreased nitric oxide bioavailability (Lesniewski et al. 2008), oxidative stress‐mediated endothelial damage (Goodwill and Frisbee 2012), increased vascular thromboxane production (Goodwill et al. 2008), augmented potassium channel signaling (Haddock et al. 2001), and vasodilatory impairments mediated by ATP‐sensitive potassium channel (Hodnett et al. 2008). Nonetheless, variability in PD contraction‐evoked vasodilation to sympathetic receptor antagonists between arteriolar orders may speak to differential Y1R‐ and *α*1R‐mediated attenuation of contraction‐evoked dilation throughout the arteriolar network. This may be a result of differences in sympathetic receptor distribution (Moore et al. 2010; Al‐Khazraji et al. 2015), differences in neural innervation density (Cowley and Franchini 1996), or differences in vascular reactivity to sympathetic receptor activation (Joshua 1991) across the vascular network. Evidently, in the current study, both 3A and 4A arterioles demonstrated greater vasoconstrictor reactivity to elevated concentrations of NPY and PE in PD compared with CTRL, where this relationship was less evident for 2A. These findings further emphasize the importance of using a “network approach” in microvascular studies and highlight the significance of considering contributions of peptidergic neurovascular control, in addition to adrenergic components, when investigating sympathetic arteriolar modulation in skeletal muscle.

As expected, combined receptor blockade recovered ROV and steady‐state vasodilatory responses in PD following all tetanic and rhythmic contractions, at all arteriolar orders studied. Interaction between Y1R and *α*1R activation has been reported, where NPY and NA act together to cause greater vasoconstriction compared to responses elicited alone (synergism), especially under conditions of increased sympathetic activation (Dahlof et al. 1985; Revington and McCloskey 1988; Jackson et al. 2005). The effect of dual Y1R and *α*1R blockade did indeed elicit the greatest increase in diameter in PD following muscle stimulation; however, the magnitude of this response was not greater than the sum of dilatory responses elicited by independent Y1R and *α*1R blockade (Jackson et al. 2005; Novielli et al. 2012), and therefore synergism between receptor types was not resolvable.

Independent of exercise, we investigated maximal arteriolar vasodilatory responses elicited by GM superfusion with SNP (10 *μ*mol/L). We observed blunting of arteriolar dilation in PD versus CTRL, where SNP‐mediated dilation of 2A and 3A were blunted by 20% and 24%, respectively. These attenuated responses to SNP application were restored following combined blockade of Y1R and *α*1R, demonstrating that elevated sympathetic receptor activation can attenuate VSMC relaxation, despite the presence of potent dilators. Contrastingly, SNP‐mediated vasodilatory responses of 4A between CTRL and PD were similar. This was not likely due to decreased 4A responsiveness to nitric oxide, since the magnitude of diameter change to SNP from baseline was similar across orders for CTRL (2A: 47%; 3A: 55%; 4A 55%). Thus, the vascular effects of nitric oxide across the arteriolar network between “*control*” and disease‐relevant models remain to be investigated.

### Emphasis on NPY‐mediated neurovascular modulation in prediabetes

This is the first study to demonstrate sympathetically mediated reductions of both ROV and steady‐state arteriolar dilation in prediabetes. In conditions such as aging and the metabolic syndrome, past studies have investigated whether decrements in functional hyperemia and vasodilation following muscle contraction were a result of enhanced *α*‐adrenergic modulation of vascular responses (Frisbee 2004; Dinenno et al. 2005; Jackson et al. 2010; Casey and Joyner 2012). In accordance with findings of the current study, past work has identified that *α*‐adrenergic receptor blockade results in increased contraction‐evoked vasodilation in aged and obese groups, and receptor activation elicits greater vasoconstrictor responses in these groups compared with controls. Additionally, a previous study demonstrated increased perfusion distribution heterogeneity of cremaster muscle arteriolar networks in obese Zucker rats (Frisbee et al. 2011). The nonspecific *α*‐adrenergic antagonist phentolamine was used to normalize perfusion distribution throughout the network to levels similar to control rats; however, perfusion distribution was only affected at 2A. To supplement findings related to adrenergic vascular modulation, investigation of peptidergic influences on perfusion distribution in the distal microcirculation would likely uncover further (and more robust) sympathetic dysregulation downstream. For example, in the rat cremaster muscle, vascular reactivity to the Y1R agonist NPY has a greater vasoconstrictor effect on distal 3A arterioles compared to proximal 1A arterioles (Joshua 1991). Notably, until currently, peptidergic modulation of contraction‐evoked vasodilation in prediabetes has not been considered. In addition to NA, it is well recognized that NPY contributes meaningfully to sympathetically mediated vascular regulation at rest, as well as during muscle contraction (Buckwalter et al. 2004, 2005; Jackson et al. 2004, 2005; Novielli et al. 2012). Under conditions of elevated SNA, neuronal NPY release and its effects on arteriolar constriction are more apparent (Bartfai et al. 1988; De Camilli and Jahn 1990; Lundberg et al. 1994). Therefore, NPY‐mediated Y1R vasoconstrictor restraint on contraction‐evoked vasodilatory responses was anticipated in prediabetic mice of this study.

## Conclusions

In this novel investigation, we demonstrated that heightened constitutive activation of Y1R and *α*1R contributes to compromised ROV and steady‐state vasodilation in response to tetanic and rhythmic muscle contractions throughout skeletal muscle arteriolar networks in prediabetic mice. Y1R and *α*1R blockade restored contraction‐evoked vasodilatory responses in PD, and Y1R and *α*1R activation attenuated contraction‐evoked vasodilatory responses in CTRL, illustrating that prediabetes is associated with greater sympathetic modulation of arteriolar function. Furthermore, arteriolar dilation elicited by topical application of SNP was attenuated in 2A and 3A of PD, where dual Y1R and *α*1R blockade in the presence of SNP normalized dilatory responses to that of CTRL levels. Finally, PD demonstrated elevated arteriolar vasoconstrictor responsiveness to topical application of increasing concentrations of NPY and PE, suggesting greater functional effects of sympathetic receptor activation in PD, or greater vascular expression of sympathetic receptors (Novielli et al. 2012). Overall, the present study provides evidence that prediabetes is associated with microvascular dysregulation related to altered sympathetic receptor activation throughout skeletal muscle branching arteriolar networks.

## Conflict of Interest

Authors have no competing interests to declare.
